# A multi-host approach to identify a transposon mutant of *Pseudomonas aeruginosa* LESB58 lacking full virulence

**DOI:** 10.1186/s13104-018-3308-7

**Published:** 2018-03-27

**Authors:** Cynthia Gagné-Thivierge, Irena Kukavica-Ibrulj, Geneviève Filion, Valérie Dekimpe, Sok Gheck E. Tan, Antony T. Vincent, Éric Déziel, Roger C. Levesque, Steve J. Charette

**Affiliations:** 1 Institut de Biologie Intégrative et des Systèmes (IBIS), Université Laval, Quebec City, QC Canada; 2 Département de biochimie, de microbiologie et de bio-informatique, Faculté des sciences et de génie, Université Laval, Quebec City, QC Canada; 3 Centre de recherche de l’Institut universitaire de cardiologie et de pneumologie de Québec, Quebec City, QC Canada; 4 Département de microbiologie, infectiologie et immunologie, Faculté de Médecine, Université Laval, Quebec City, QC Canada; 5 INRS-Institut Armand Frappier, Laval, QC Canada

**Keywords:** *Pseudomonas aeruginosa*, Cystic fibrosis, Lung infection, Virulence factors, LESB58, Rat, *Dictyostelium discoideum*, *Drosophila melanogaster*

## Abstract

**Objective:**

*Pseudomonas aeruginosa* is an opportunistic bacterial pathogen well known to cause chronic lung infections in individuals with cystic fibrosis (CF). Some strains adapted to this particular niche show distinct phenotypes, such as biofilm hyperproduction. It is necessary to study CF clinical *P. aeruginosa* isolates, such as Liverpool Epidemic Strains (LES), to acquire a better understanding of the key genes essential for in vivo maintenance and the major virulence mechanisms involved in CF lung infections. Previously, a library of 9216 mutants of the LESB58 strain were generated by signature-tagged mutagenesis (STM) and screened in the rat model of chronic lung infection, allowing the identification of 163 STM mutants showing defects in in vivo maintenance.

**Results:**

In the present study, these 163 mutants were successively screened in two additional surrogate host models (the amoeba and the fruit fly). The STM PALES_11731 mutant was the unique non-virulent in the three hosts. A competitive index study in rat lungs confirmed that the mutant was 20-fold less virulent than the wild-type strain. This study demonstrated the pertinence to use a multi-host approach to study the genetic determinants of *P. aeruginosa* strains infecting CF patients.

**Electronic supplementary material:**

The online version of this article (10.1186/s13104-018-3308-7) contains supplementary material, which is available to authorized users.

## Introduction

*Pseudomonas aeruginosa* is one of the most common pathogenic bacteria causing lung infections among cystic fibrosis (CF) patients [1]. *P. aeruginosa* infecting CF patients undergo microevolution: mucoid strains, which also express lower levels of virulence factors such as type three secretion system (TTSS) effectors, are favored. Those are the CF-adapted *P. aeruginosa* strains [2, 3].

The Liverpool Epidemic Strain (LES) B58 (LESB58) is one of those strains found in chronic CF lung infections and one of the first *P. aeruginosa* strains identified as epidemic among CF patients [4]. The phenotypic features of LES include biofilm hyperproduction and resistance to several clinically useful antibiotics [5].

*Pseudomonas aeruginosa* studies classically use the strain PAO1, originally isolated from a human wound [6]. However, knowing that CF-adapted strains have unique phenotypes, it is necessary to use CF-adapted strains such as LESB58 to explore genes involved in the virulence and the in vivo maintenance of *P. aeruginosa* in CF lungs.

To determine which genes are the most important for LESB58’s pathogenicity, signature-tagged mutagenesis (STM) was used to create 9216 mutants. In a screening for the survival of the STM mutants in the rat model of chronic lung infection, 163 mutants had a growth defect in vivo, suggesting subdued virulence [7].

In the present study, we performed additional screening of the 163 mutants, this time using successively the amoeba *Dictyostelium discoideum* and the fly *Drosophila melanogaster* as two other surrogate host models. The rat, the amoeba and the fly are three very different model hosts in the study of bacterial virulence and served respectively as a chronic lung infection model [8], a phagocyte model [9], and a systemic infection model [10]. We were able to identify that the STM PALES_11731 mutant was the only one that was defective in all three hosts.

## Main text

### Materials and methods

#### Bacterial strains

The *P. aeruginosa* LESB58 strain was isolated from a chronic lung infection of a CF patient in Liverpool (United Kingdom), in 1996 [4]. We later reported its STM PALES_11731 mutant, in 2009 [7].

#### *Dictyostelium discoideum* predation assay

Bacterial lawns were prepared by suspending bacteria in lysogeny broth (LB) (OD_595_ of 2) and spreading 300 μL of this suspension on a SM 1/5 Petri dish [11]. *D. discoideum* DH1-10 cells routinely grown at 21 °C in HL5 medium supplemented with 15 μg/mL of tetracycline were used as a host [12]. Cells were washed and resuspended in HL5 without tetracycline, counted using a haemocytometer, and serial dilutions were performed to obtain: 3000, 1000, 300, 100, 30 or 10 cells/5 μL. These dilutions were spotted (5 μL drops) on the dried bacterial lawn and the Petri dishes were incubated at room temperature (21–23 °C) for 6 days.

#### Fly pricking assay

Adult female flies aged of 7 ± 2 days were pricked according to a modified previously published protocol [13]. Bacterial cells were grown in tryptic soy broth (TSB) and diluted to an OD_600_ of 0.2 in a sterile solution of 10 mM MgSO_4_ supplemented with 100 μg/mL ampicillin. The flies were anesthetized using CO_2_ and pricked in the dorsal thorax using a 23S gauge Hamilton needle dipped in the appropriate bacterial suspension. For each strain tested, at least 30 flies were infected. The flies were separated into groups of 10 in vials containing 5% sucrose solidified with 1.5% agar. At least 10 control flies were also pricked with a solution of 10 mM MgSO_4_ supplemented with 100 μg/mL ampicillin. The flies were kept at 25 °C and 65% humidity. Fly survival was recorded daily and survival data was compiled and analyzed with Kaplan-Meier survival curves. The log-rank (Mantel–Cox) test was used to assess significance between the curves.

#### Competitive index in rat model

Agar beads were prepared according to a modification of a previously described method [8, 14]. The STM PALES_11731 mutant (tagged with tetracycline resistance within mini-Tn*5* transposon) and the wild-type LESB58 were grown separately in TSB. Overnight cultures were sedimented by centrifugation (3000×*g*, 10 min), washed twice with 1 mL of phosphate buffered saline (PBS), and added to 9 mL of 2% agar, prewarmed to 48 °C. A mixture of equal counts of wild-type and mutant cells was added to 200 mL heavy mineral oil at 48 °C with rapid stirring on a magnetic stirrer in a water bath for 5 min at room temperature, followed by 10 min without stirring. The oil-agar mixture was centrifuged (3000×*g*, 20 min) to sediment the beads and washed twice with PBS. The preparations, containing beads of 100–200 µm in diameter, were used as inocula for animal experiments. The number of bacteria in the beads was determined by homogenizing the bacterial bead suspension and plating 10-fold serial dilutions on Mueller–Hinton agar (MHA) and MHA supplemented with 45 µg/mL tetracycline.

Six Sprague–Dawley rats were anaesthetized using isofluorane (2% of respiratory volume) and inoculated by intubation using a venous catheter 18G and syringe (1-cc Tuberculin) with 120 µL of a suspension of agar beads-embedded bacteria containing approximately 2 × 10^7^ colony-forming units (CFU)/injection. 7 days later, the bacteria were extracted from the infected rat lungs and counted using MHA for the total bacterial number of LESB58 wild-type cells and STM mutant cells or with MHA with 45 µg/mL tetracycline for STM mutant selection.

The in vivo competitive index (CI) was determined as the CFU output (in vivo) ratio of the STM PALES_11731 mutant in comparison to the wild-type strain, divided by the CFU input ratio of mutant to wild type [15, 16]. The final CI was calculated as the geometric mean of the individual animals’ CI.

### Results and discussion

#### Reduced virulence of LESB58 mutants in *Dictyostelium discoideum*

**Fig. 1:**
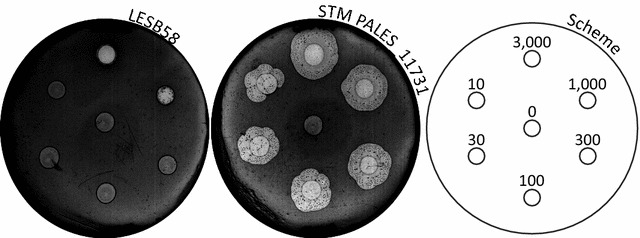
Example of a non-virulent LESB58 STM mutant in the amoeba model. Drops of determined amoeba concentrations were spotted on a bacterial lawn, allowing a semi-quantitative determination of the bacteria’s predation resistance after 6 days of incubation at 21–23 °C. Phagocytic plaques appear as light areas on the bacterial lawn. At least 1000 *D. discoideum* cells/5 μL were required to pierce the wild-type bacterial lawn, whereas fewer amoeba cells were needed to form a phagocytic plaque for mutants with reduced virulence in this context. The results were later confirmed (n = 3)

We assessed the ability of the 163 STM mutants identified in the rat lung infection model [7] to resist the predation of *D. discoideum* cells, a well-recognized model to study the virulence of *P. aeruginosa* [9, 11, 17]. The phagocytosis mechanism in *D. discoideum* highly resembles that of human macrophages [18]. This host therefore allows the identification of bacteria able to kill phagocytes or to resist to their internalization or digestion [17]. Among the 163 mutants, 45 mutants displayed sensitivity to amoeba predation. Fourteen of them were highly sensitive, as revealed specifically by the formation of large phagocytic plaques for any given concentration of *D. discoideum* cells on the lawn of these mutants, whereas only the highest amoeba concentrations could produce small phagocytic plaques when using the wild-type bacterium (Fig. 1). Three of the mutants had a growth defect assessed with Bioscreen C (data not shown). Therefore, only the remaining 11 mutants, previously selected in the rat model and sensitive to predation in the amoeba model (Additional file 1: Table S1), were kept for further analysis in the *Drosophila* host model.

#### Identification of the STM PALES_11731 mutant with the *Drosophila* systemic model

**Fig. 2:**
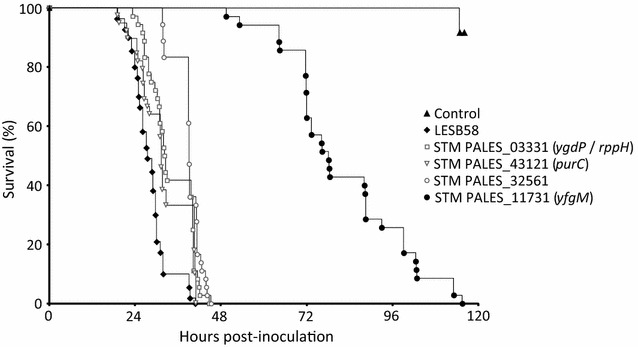
The STM PALES_11731 mutant has a very low virulence in the drosophila infection model. At least 30 flies were pricked with a needle dipped in bacterial suspension and the insect survival rate was followed over time. By comparison with the wild-type strain (black diamonds), which caused the death of infected flies in less than 40 h, 4 of the 11 mutants were less virulent. One of them (STM PALES_11731 mutant, black circles) was particularly less virulent, with some flies surviving the infection until about 115 h. For the sake of clarity, the 7 mutants displaying virulence equivalent to the wild-type strain (LESB58) are not shown on the graph

Because the well-studied immune system of *D. melanogaster* shares similarities with that of mammals, this host provides an easy alternative model of infection for the study of human pathogens’ virulence mechanisms [19, 20]. *D. melanogaster* can serve as a model host for systemic *Pseudomonas* infections [10] and was used as a third surrogate model to identify LESB58 mutants with a broad virulence defect, resulting in them being less able to cause systemic infections. The test was performed using the 11 remaining STM mutants. Four of these mutants were less virulent in this assay, as the flies’ survival time was significantly longer when compared to the wild type (Fig. 2). However, the STM PALES_11731 mutant was by far the most attenuated, with a survival time 80 h longer than LESB58, compared to only a delay of 1–5 h for the three other reduced mutants.

#### The STM PALES_11731 mutant is strongly attenuated in the chronic lung infection model

**Fig. 3:**
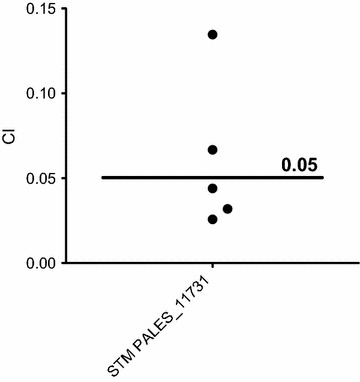
The STM PALES_11731 mutant is 20 times less virulent than the wild-type bacteria in a rat lung infection model. Rats were infected with a mixture of mutant and wild-type cells in an equal ratio. After 7 days, CFU recovered for the mutant compared to the wild-type bacteria were calculated to obtain the competitive index (CI). Because one rat died before the end of the experiment, only the results for five of the six rats were analyzed. Each circle represents the CI determined in each rat. A CI < 0.4 indicates a highly attenuated persistence of the mutant [21]. The mean CI for the STM PALES_11731 mutant is 0.05 and, knowing that it is not due to a growth defect (see Additional file 1: Figures S6 and S7), these results confirm that STM PALES_11731 mutant virulence is seriously compromised in this model

To estimate the defect in virulence of the STM PALES_11731 mutant, a CI in combination with the wild-type strain was performed in the rat lung model. The CI allows a quantitative evaluation of the defect for in vivo maintenance in a model of chronic lung infection [21]. A CI of 0.05 was obtained for the STM PALES_11731 mutant (Fig. 3), indicating that the mutant is 20-fold less capable of in vivo maintenance than the wild-type strain. Considering this, the STM PALES_11731 mutant was further characterized to identify the mutated gene and its potential role in the virulence of LESB58.

#### Potential consequences of the transposon insertion in the STM PALES_11731 mutant

Genotyping [7] of the STM PALES_11731 mutant revealed that the mini-Tn5-*tet* transposon was inserted in the 3′ region of gene PALES_11731 (*yfgM*), 10 nucleotides before the end of the gene’s sequence (Additional file 1: Figure S1). PALES_11731 codes for YfgM, an ancillary SecYEG translocon subunit [22] for which the exact function remains unclear. The insertion introduces a stop codon and the last two amino acids of the translated protein are missing (Additional file 1: Figure S2). The predicted structure of the protein suggests that the mutation does not clearly affect the YfgM function. In *Escherichia coli*, an inactivation of *yfgM* increases the bacteria sensitivity to acidity [22]. The STM PALES_11731 mutant resistance to acid stress was, however, the same as for the wild-type strain (Additional file 1: Table S2), supporting the idea that YfgM is still functional.

Polar effects are frequently observed in transposon mutagenesis. The mutated operon contains nine genes and the transposition in *yfgM*, the seventh, could have silenced the two downstream genes. RT-qPCR confirmed that there was no significant transcription defect of these genes (Additional file 1: Figure S3). The following phenotypic tests were performed to confirm this result.

The eighth gene of the operon is PALES_11741 (*bamB*, previously named *yfgL*), coding for the outer membrane protein assembly factor BamB (YfgL). In *Salmonella enterica*, *bamB* was found to be necessary for the expression of the TTSS [23], a major virulence mechanism for many bacteria, including *P. aeruginosa* [24–26]. We tested the TTSS expression of the STM PALES_11731 mutant but no significant difference with the wild type could be observed (Additional file 1: Figure S4).

It was demonstrated that a *bamB* mutant in reference strain PAO1 is highly sensitive to antibiotics (especially those targeting cell wall synthesis) and lysozymes [27]. Therefore, we tested the resistance of the STM PALES_11731 mutant to piperacillin (a β-lactam antibiotic), tobramycin (an aminoglycoside antibiotic) and lysozymes. There was no significant difference with the wild type (Additional file 1: Figure S5). This is an additional indication that *bamB* might not be affected by the transposition in the STM PALES_11731 mutant.

The ninth gene of the operon is *engA* (also known as *der*), coding for the GTP-binding protein EngA (GTPase Der). This protein plays an essential role in ribosome biogenesis and its inactivation causes growth defects, especially at cold temperatures [28]. Considering this, we compared the STM PALES_11731 mutant and the wild type growth in different conditions. LB medium, a not-restrictive medium, was chosen as it is the most commonly used on *P. aeruginosa*. SM 1/5 medium, a diluted low-nutrient medium, was also tested. This medium was used for the amoeba predation assay at 21–23 °C. In LB medium at 37 °C, the wild-type strain, but not the STM PALES_11731 mutant, formed floating aggregates resembling biofilm. Since the usual biofilm definition implies an adhesion to a surface, we can refer to these unattached structures as biofilm-like structures (BLSs) [29]. This phenomenon caused variability in the growth curve measurements, but could be inhibited by the addition of Mg^2+^. Growth in LB medium supplemented with MgCl_2_ confirmed the absence of a growth defect for the STM PALES_11731 mutant (Additional file 1: Figure S6). The mutant growth was also comparable to the wild-type LESB58 in SM 1/5 medium at 21 °C (Additional file 1: Figure S7), which shows that *engA* is likely expressed in the mutant strain. These results also indicated that the STM PALES_11731 mutant defect in virulence is not due to an inherent growth defect.

#### The highly reduced virulence of the STM PALES_11731 mutant is likely multifactorial

The absence of a BLS formation for the STM PALES_11731 mutant, despite a similar-to-the-wild-type adhered biofilm formation (Additional file 1: Figure S8), cannot itself completely explain the multi-host lack of virulence in the mutant. The amoeba predation assay was performed in a condition in which the wild-type strain does not form BLSs (Additional file 1: Figure S7). However, there was a clear lack of resistance to phagocytosis of the mutant compared to the wild-type strain (Fig. 1). Thus, there must be one or several other virulence mechanisms defective in the mutant to explain its weak resistance to amoeba predation.

Future studies will be necessary to fully understand the impact of the mini-Tn*5*-*tet* transposon insertion in the operon containing *yfgM*. Considering the broad virulence defect of the STM PALES_11731 mutant, this operon appears to play a key role in LESB58 virulence.

### Limitations

Because the transposon did not appear to have an impact on the expression of any of the analyzed genes, it was not possible to link the lack of virulence of the STM PALES_11731 mutant with a specific gene.

## Additional file

**Additional file 1.** All additional protocols, figures and tables cited in this article.

## Abbreviations

BLS: biofilm-like structure
CF: cystic fibrosis
CFU: colony-forming unit
CI: competitive index
LB: lysogeny broth
LES: Liverpool Epidemic Strain
MHA: Mueller–Hinton agar
OD: optical density
PBS: phosphate buffered saline
rpm: revolutions per minute
RT-qPCR: reverse transcription quantitative polymerase chain reaction
STM: signature-tagged mutagenesis
TSB: tryptic soy broth
TTSS: type three secretion system
