# Supplementary material for: A multi-host approach to identify a transposon mutant of Pseudomonas aeruginosa LESB58 lacking full virulence
Source: BMC Res Notes. 2018 Mar 27;11:198. doi: 10.1186/s13104-018-3308-7 (PMC5870910; doi:10.1186/s13104-018-3308-7)
Supplement: Supplementary file 1 — Additional file 1. All additional protocols, figures and tables cited in this article. [file 13104_2018_3308_MOESM1_ESM.docx]

ADDITIONAL FILES, TABLE OF CONTENTS

**TITLE: A multi-host approach to identify a transposon mutant of *Pseudomonas aeruginosa* LESB58 lacking full virulence**

**AUTHORS:** Cynthia Gagné-Thivierge, Irena Kukavica-Ibrulj, Geneviève Filion, Valérie Dekimpe, Sok Gheck E Tan, Antony T. Vincent, Éric Deziel, Roger C. Levesque and Steve J. Charette

[Table S1. 1](#_Toc505003202)

[Protocol for acid-stress assay 2](#_Toc505003203)

[Table S2. 2](#_Toc505003204)

[Figure S1. 3](#_Toc505003205)

[Figure S2. 4](#_Toc505003206)

[Protocol for RT-qPCR 5](#_Toc505003207)

[Table S3. 5](#_Toc505003208)

[Figure S3. 7](#_Toc505003209)

[Protocol for induction of TTSS and secretome evaluation 8](#_Toc505003210)

[Figure S4. 9](#_Toc505003211)

[Protocol for resistance of LESB58 and the STM PALES_11731 mutant to antibiotics and lysozyme 10](#_Toc505003212)

[Figure S5. 10](#_Toc505003213)

[Figure S6. 12](#_Toc505003214)

[Figure S7. 13](#_Toc505003215)

[Figure S8 14](#_Toc505003216)

[REFERENCES 15](#_Toc505003217)

Table S1. **List of non-virulent mutants in the amoeba predation assay and tested in the *Drosophila* model of infection.** 11 of the 14 mutants that are avirulent against *D. discoideum* were tested against *D. melanogaster*. These mutants and their corresponding inactivated gene are listed here.

| **Mutants** | **STM insertion location** | **Reference in PAO1** | **Corresponding altered protein** |
| --- | --- | --- | --- |
|  |  |  |  |
| L52T5T | PALES_23991 | PA2705 | Hypothetical protein |
| L54T13T | PALES_43701 (*purM*) | PA0945 | Phosphoribosylformylglycinamidine cyclo-ligase |
| L70T18G | PALES_03331 (*ygdP* / *rppH*) | PA0336 | RNA pyrophosphohydrolase |
| L137T1G | PALES_43121 (*purC*) | PA1013 | Phosphoribosylaminoimidazole-succinocarboxamide synthase |
| L138T21T | PALES_32561 | PA2066 | Hypothetical protein |
| L149T6G | PALES_19521 (*purF*) | PA3108 | Amidophosphoribosyltransferase |
| L154T16G | PALES_11981 | PA3776 | Putative transcriptional regulator |
| L155T7T | PALES_11731 (*yfgM*) | PA3801 | Ancillary SecYEG translocon subunit |
| L162T19T | PALES_21911 (*tgpA*) | PA2873 | Protein-glutamine gamma-glutamyltransferase |
| L166T22G | PALES_07851 | PA4142 | Putative secretion protein |
| L173T2G | PALES_31031 | None* | FadE36, putative aminoglycoside phosphotransferase |

*Not present in PAO1, but present in PA14 (PA14_35990).Protocol for acid-stress assay

We used a modified version of the protocol from Götzke *et al.*.[1] Bacteria were suspended (OD_595_ = 0.1) in 5×3 mL of LB medium and incubated at 37°C, 200 rpm, for 4 hours. The 5 cultures were pooled (15 mL total), then separated in 3 tubes (5 mL/tube), one for each of the tested pH. After centrifugation (3220 × *g*, 10 min) to pellet the cells, the bacteria were resuspended in 5 mL of LB medium at pH 7 (control condition), pH 4 or pH 2. Following an incubation of 30 min at 37°C, 200 rpm, the cultures were centrifuged (3220 × *g*, 10 min) and the bacteria were resuspended in sterile water (DO_595_ = 0.2). Serial dilutions (10^0^ to 10^-7^) were spotted as 5 μL drops on LB agar. Results were obtained after 40 hours of incubation at 37°C.

Table S2. **Bacterial growth after treatment at a low pH.** Following acid-stress treatment, the STM PALES_11731 mutant and the wild-type cells were grown on LB agar. There was no difference between the strains (n=3).

|  | **Dilutions** | | | | | | | | **pH** |
| --- | --- | --- | --- | --- | --- | --- | --- | --- | --- |
|  | **10^0^** | **10^-1^** | **10^-2^** | **10^-3^** | **10^-4^** | **10^-5^** | **10^-6^** | **10^-7^** |  |
| **LESB58** | +++ | +++ | +++ | +++ | ++ | + | – | – | **7** |
|  | +++ | +++ | +++ | ++ | + | + | – | – | **4** |
|  | – | – | – | – | – | – | – | – | **2** |
| **STM PALES_11731** | +++ | +++ | +++ | +++ | ++ | + | – | – | **7** |
|  | +++ | +++ | +++ | ++ | + | + | – | – | **4** |
|  | – | – | – | – | – | – | – | – | **2** |

**
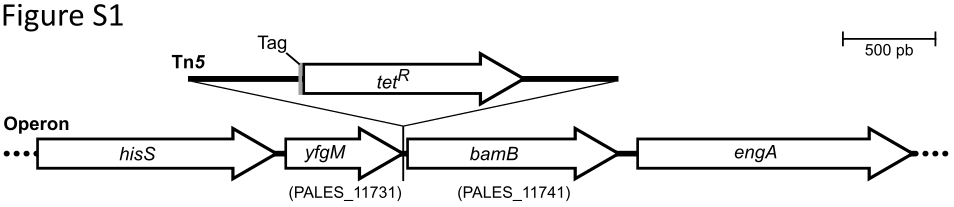
**

Figure S1. **Representation of the mini-Tn*5*-*tet* transposon insertion in the STM PALES_11731 mutant.** The transposon, containing a tetracycline resistance gene, is inserted 10 nucleotides before the end of the *yfgM* gene. For the sake of clarity, only the last 4 genes of the operon (also corresponding to the genes later on tested in RT-qPCR (**additional files fig. S3**)) are shown.

Figure S2. **Structural analyses of the protein encoded by the gene PALES_11731.** (**A**) The primary sequence of the protein is in bold and the predicted disordered residues are in blue. The topology with respect to the membrane, as predicted by TOPCONS[2], is indicated for each residue (i: inside the membrane, o: outside of the membrane, m: membrane region). (**B**) Region where the transposon mini-Tn*5*-*tet* is inserted. Precisely, it inserted between the second and third nucleotides of the codon for the glycine at position 212. The nucleotides from the mini-Tn*5*-*tet* are in red. (**C**) 3D structure predicted using I-TASSER 5.1[3] of the residues from the protein encoded by PALES_11731 that are predicted to be outside the membrane (42 to 214). The glutamic acid (position 213) and the alanine (position 214) that are missing due to the insertion of the transposon are in green. The best predicted model has a C-score of 0.02 and a TM-score of 0.72.

# Protocol for RT-qPCR

Bacteria were suspended (OD_595_ = 0.1) in 3 mL of LB medium and incubated at 37°C, 200 rpm. To prevent BLS formation, which begins in the late exponential growth phase (see **additional files fig. S6A**), bacterial growths were performed for a short period of time (4 hours) in 4 tubes for each replicate. The 4 cultures were pooled (12 mL total) and bacteria concentrated by centrifugation (3220 × *g*, 10 min) and resuspension in 1 mL of fresh medium. RNA extraction was performed using the RiboPure™ RNA Purification Kit for bacteria (ThermoFisher) as suggested by the manufacturer. Extracted RNA was treated with DNase I and purity was assessed by electrophoresis on 1% agarose gel. Retrotranscription was performed on the total extracted RNA using the iScript™ Advanced cDNA Synthesis Kit for RT-qPCR (Bio-Rad) according to the instructions of the manufacturer. qPCR was achieved in Rotor-Gene Q (Qiagen) with SYBR® Select Master Mix (ThermoFisher) using *gyrB* as the reference housekeeping gene. RNA extraction and retrotranscription were performed in independent duplicates for each bacteria and each sample obtained was tested in duplicates qPCR reactions. For each gene’s standard curve, R^2^ > 0.99.

Table S3. **List of primers.** Primers used for PCR amplification of genes templates and qPCR of the cDNA obtained to measure the expression of those genes.

|  | **Target** | **Primer** | **Sequence (5' -> 3')** | **Product length (pb)** |
| --- | --- | --- | --- | --- |
| Template amplification in PCR | *gyrB* | gyrB_FWD | CATGAACTACGCCTGACCAT | 520 |
|  |  | gyrB_REV | CTTCGGCCTCGATGTAGTTG |  |
|  | *hisS* | hisS_FWD | AAACCGCAGAAAGGTCGCTA | 483 |
|  |  | hisS_REV | AAGCTTGTCGGTGACCCATT |  |
|  | *yfgM* (PALES_11731) | yfgM_FWD | GCGTACCGAAGACGAAGA | 278 |
|  |  | yfgM_REV | TGCCGTACTGGGCATAAT |  |
|  | *bamB* (PALES_11741) | bamB_FWD | GGCAAGAAGAAGTGGACCAAG | 600 |
|  |  | bamB_REV | CACCACGTTGCTGGAGAACA |  |
|  | *engA* | engA_FWD | ACAGCATCTACATCCCGTTC | 694 |
|  |  | engA_REV | GAGTTCTTCTTGCCCTCGTAG |  |
| Amplification in qPCR | *gyrB* | qPCRgyrB_FWD | GAGCTGTCCTTCCTCAACTC | 92 |
|  |  | qPCRgyrB_REV | TTCAGACCGCCTTCGTACTT |  |
|  | *hisS* | qPCRhisS_FWD | AAGAATCGATCGCCCACTTC | 109 |
|  |  | qPCRhisS_REV | GTGCGGCAGTAATAGTCCAG |  |
|  | *yfgM* (PALES_11731) | qPCRyfgM_FWD | CTACCAGCAACTGATCGAAACC | 95 |
|  |  | qPCRyfgM_REV | CGAACTCGCTCTTCAGCTTG |  |
|  | *bamB* (PALES_11741) | qPCRbamB_FWD | GAAGGCTTCGGCAATATCTAC | 87 |
|  |  | qPCRbamB_REV | GTTGTTCCACAGCGAAGAAG |  |
|  | *engA* | qPCRengA_FWD | GTCATTGCCCTGAACAAGTG | 118 |
|  |  | qPCRengA_REV | GGGCCGAAATGAAATGGATG |  |

**
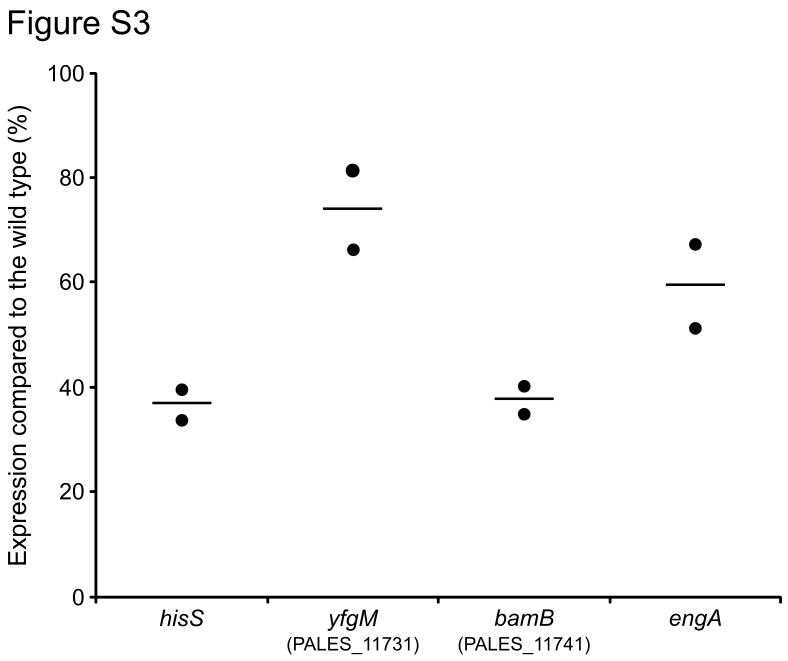
**

Figure S3. **Percentage of expression of the genes *hisS*, *yfgM*, *bamB* and *engA* in the STM PALES_11731 mutant relatively to the wild-type strain.** Quantification of the expression of the genes surrounding the mini-Tn*5*-*tet* transposon insertion site (see **additional files fig. S1**) was performed using RT-qPCR and compared to the wild-type strain’s expression of those genes. The housekeeping gene *gyrB* was used as a reference. The difference of expression in the STM PALES_11731 is illustrated as a percentage of expression relatively to the expression in the wild-type strain. Each circle represents the average result of two technical replicates on one biological replicate. The horizontal line is the mean for the two biological replicates for each gene.

# Protocol for induction of TTSS and secretome evaluation

Protocol modified from Alibaud *et al.*.[4] Bacteria were suspended (OD_595_ = 0.1) in 4 mL of LB medium supplemented or not with 5 mM EGTA and 20 mM MgCl_2_ to induce TTSS expression. After overnight (18 hours) at 37°C, 200 rpm, the supernatant of each culture was recovered by two successive centrifugations (10,000 × *g*, 10 min), followed by one filtration with a filter with 0.2 μm pores (Filtropur S 0.2, Sarstedt Canada). The proteins contained in the supernatant were then concentrated by about 60-fold by passing a total of 3 mL of supernatant through a Nanosep centrifugal device (Nanosep 3K Omega, Pall Life Sciences) as suggested by the manufacturer (centrifugations used : 14,000 × *g*, 17 min). About 50 μL of liquid was recovered and the volume adjusted with fresh medium, so that all samples had the same final volume. 30 μL of each concentrated sample was mixed with 15 μL of denaturation solution (TEX 3X + 15% β-Mercaptoethanol) and heated to 95°C for 5 min. Finally, 15 μL of each sample were analyzed on a 12% SDS-PAGE gel and visualized by Coomassie blue staining.

The proteins in the control or TTSS-inducing conditions were identified by mass spectrometry. The percentage of total spectra obtained that corresponded to identified TTSS related proteins (PopB, PcrV, YopN, PopD, ExoT, ExoY, PscF, PscP and HopJ) found in supernatant was calculated. To quantify the induction effect on the TTSS protein production, the percentage obtained in the TTSS-inducing conditions was divided by the percentage obtained in the control conditions for each strain. Because the percentage of total spectra corresponding to a protein is related to the total quantity of proteins present in the sample, the calculated induction was corrected by the ratio of [total number of proteins in TTSS-inducing conditions / total number of proteins in control conditions].

**
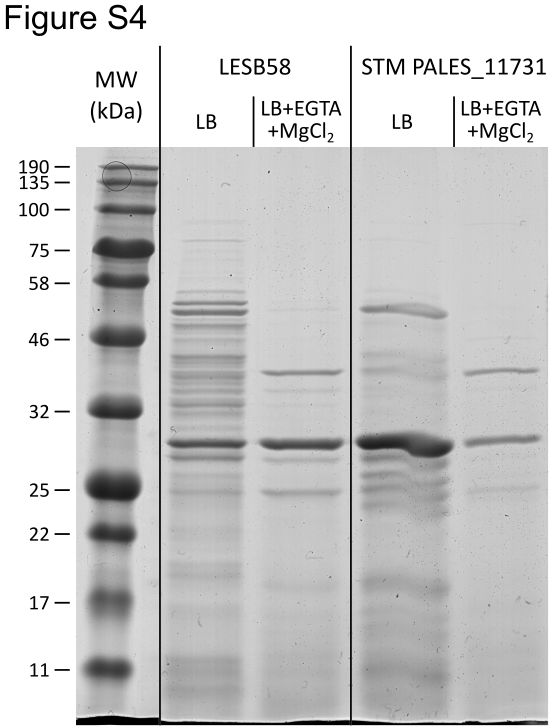
**

Figure S4. **Protein contents of the wild-type strain LEBS58 and the STM PALES_11731 mutant supernatant visualized by SDS-PAGE 12%.** The experiment has been performed 3 times with similar results, and one representative experiment is shown. Proteins obtained in both conditions were identified by mass spectrometry. For both the wild-type and the mutant, it was possible to observe a higher relative proportion of TTSS-related proteins under inducing conditions (a relative proportion of 4.31 in the mutant and 8.10 in the wild-type). Based on the results of the SDS-PAGE and mass spectrometry analyses for the TTSS proteins, induction of protein secretion was likely similar (in terms of which TTSS proteins were secreted and the relative proportion of all TTSS proteins found in the supernatant), indicating that the transposition in the STM PALES_11731 mutant did not significantly affect the TTSS expression.

# Protocol for resistance of LESB58 and the STM PALES_11731 mutant to antibiotics and lysozyme

For the antibiotic sensitivity assay, bacteria suspended (OD_595_ = 2) in 300 μL of Müller-Hinton broth (MHB) were spread on a Müller-Hinton agar (MHA) Petri dish to form a bacterial lawn. Paper disks containing either piperacillin (30 or 100 μg) or tobramycin (10 or 30 μg) were gently placed on the dried bacterial lawn. Results were obtained after overnight growth at 37°C. Lysozyme from chicken egg white (Sigma-Aldrich) was used for the lysozyme sensitivity assay to the following concentrations: 0.5, 1 and 2 mg/mL. Bacteria were suspended (OD_595_ = 0.1) in 300 μL of LB medium in 48-well plates in triplicate and incubated in a microplate reader (Infinite 200Pro NanoQuant TECAN) at 37°C for 20 h.

**
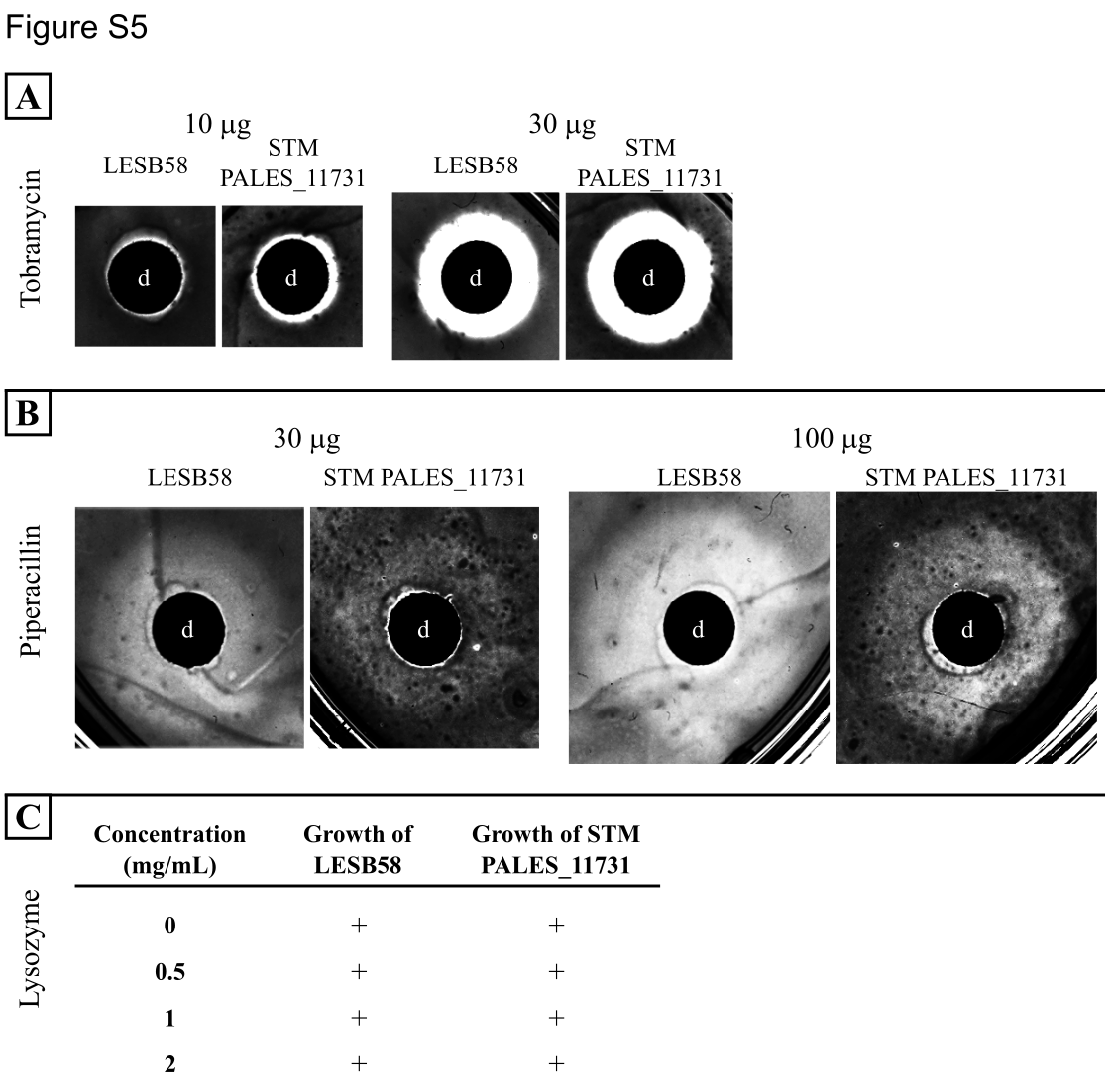
**

Figure S5. **The STM PALES_11731 mutant shows the same antibiotic and lysosyme resistance as the wild-type LESB58.** The sensitivity of the bacteria to (**A**) tobramycin and (**B**) piperacillin is represented by the zone of inhibition (clear zone) created on the bacterial lawn around the disks (d). There was no difference between the STM PALES_11731 mutant and the wild-type strain with the two antibiotics tested (n=3). In **C**, growth of the bacteria is shown by a plus (+) and a lack of growth is shown by a minus (–). The resistance to the lysozyme activity was similar for both strains (n=3).


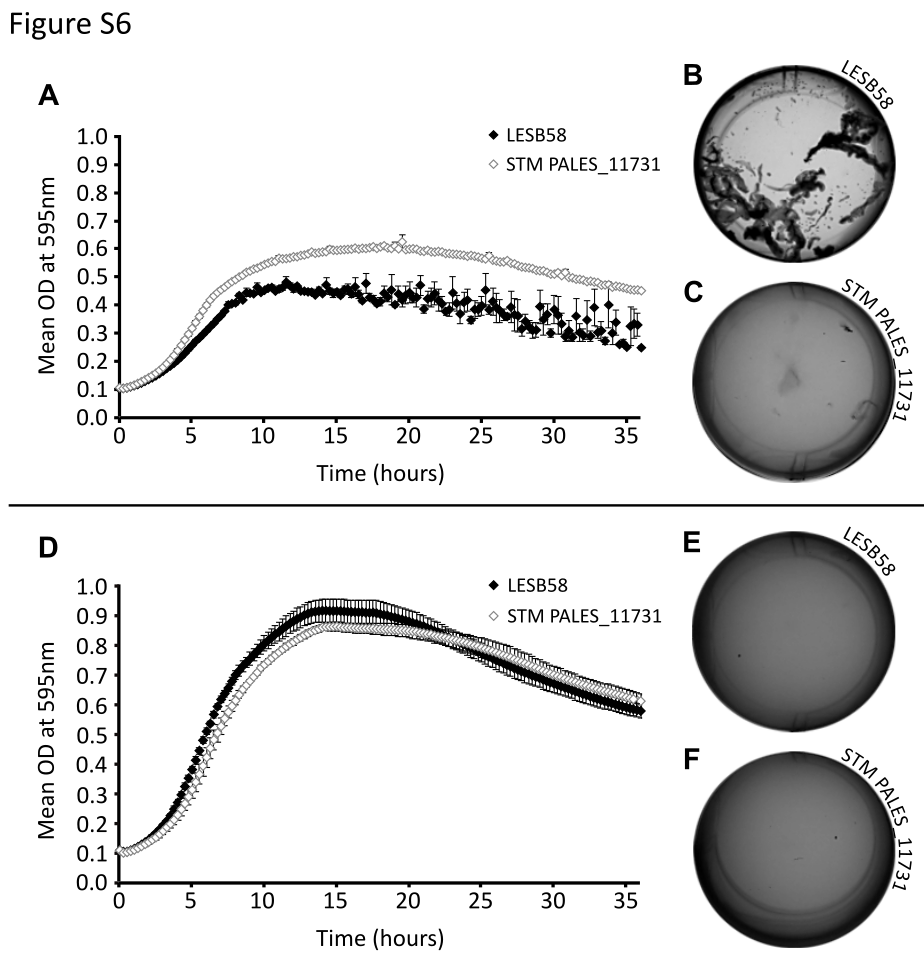


Figure S6. **Similar growth of wild-type LESB58 and STM PALES_11731 mutant strains when BLSs are inhibited by magnesium ions.** Growth curves of LESB58 (black) and the STM PALES_11731 mutant (white) were established in a microplate reader at 37°C in a 24-well plate. **A** In LB medium, the LESB58 strain forms biofilm-like structures (BLSs) (see **B**) whereas the STM PALES_11731 mutant does not (see **C**). The means were obtained from 9 separate experiments. (**D**) In a medium intended to inhibit the formation of BLSs (LB medium supplemented with 20 mM MgCl_2_), no BLSs are formed (see **E** (LESB58) and **F** (STM PALES_11731 mutant)) and the growth was similar for both bacteria (p-value = 0.45). Means were obtained from the data of 3 separate experiments. Error bars show the calculated standard deviation of the mean. The growth curves were compared using the function compareGrowthCurves of the R package statmod.[5]


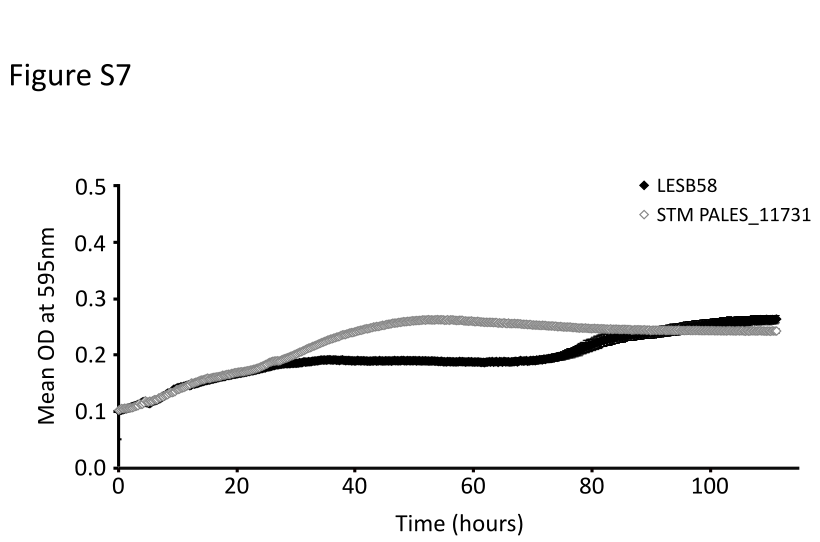


Figure S7. **Low nutrient medium does not hinder the mutant growth.** The growth curves of LESB58 (black) and the STM PALES_11731 mutant (white) in SM 1/5 medium at 21°C were established in a microplate reader for a 24-well plate. The mutant shows an initial growth better than the wild-type strain. The data was averaged over 4 separate experiments. Errors bars show the calculated standard deviation of the mean.


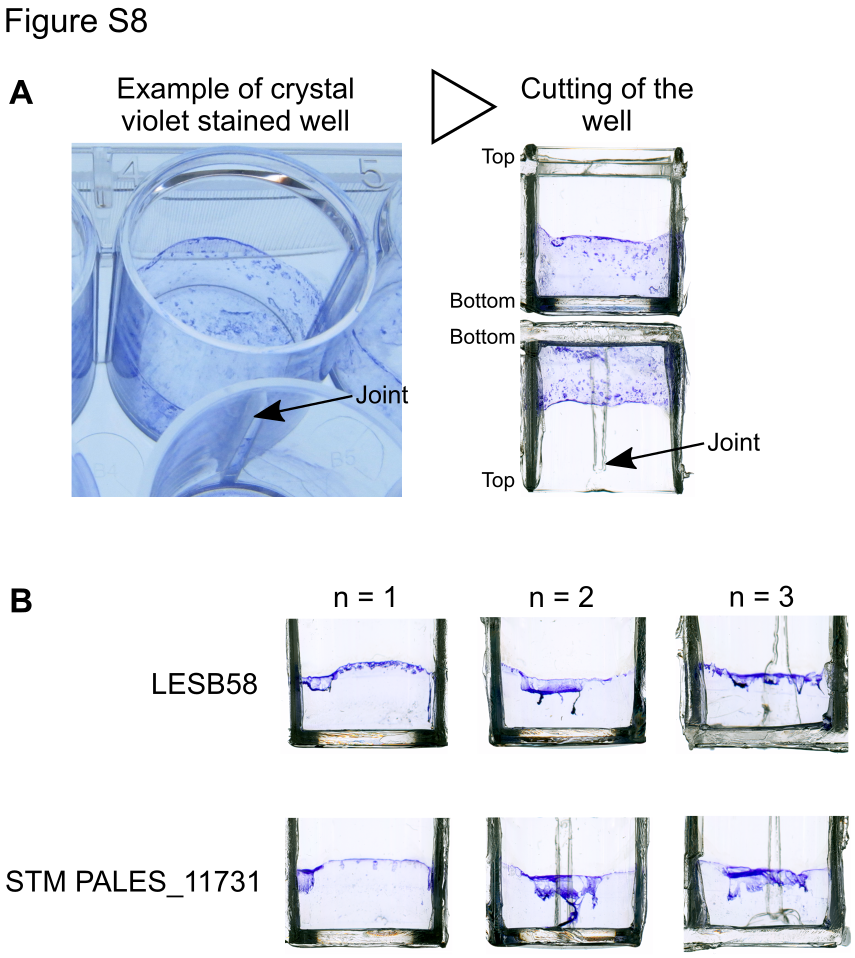


Figure S8**. Similar adhered biofilm formation of wild-type LESB58 and STM PALES_11731 mutant strains.** The bacteria were grown during 24 hours at 37°C, 200 rpm, in a 24-well plate (600 μL per well). (**A**) Modified from a previously published protocol [6], the wells were emptied, washed twice with 0.9% NaCl sterile saline, and the biofilms were stained with a crystal violet solution (0.1% in water). After two subsequent washings (0.9% NaCl sterile saline), the wells were dried at room temperature, cut in half and scanned (HP Scanjet G4050). (**B**) The amount of biofilm that formed on the well’s wall is equivalent in the STM PALES_11731 mutant and the wild-type strain (n=3).

# REFERENCES

1. Gotzke H, Muheim C, Altelaar AFM, Heck AJR, Maddalo G, Daley DO. Identification of putative substrates for the periplasmic chaperone YfgM in *Escherichia coli* using quantitative proteomics. Mol Cell Proteomics. 2015;14:216–26.

2. Tsirigos KD, Peters C, Shu N, Kall L, Elofsson A. The TOPCONS web server for consensus prediction of membrane protein topology and signal peptides. Nucleic Acids Res. 2015;43:W401-7.

3. Yang J, Yan R, Roy A, Xu D, Poisson J, Zhang Y. The I-TASSER Suite: protein structure and function prediction. Nat Meth. 2015;12:7–8.

4. Alibaud L, Köhler T, Coudray A, Prigent-Combaret C, Bergeret E, Perrin J, et al. *Pseudomonas aeruginosa* virulence genes identified in a *Dictyostelium* host model. Cell Microbiol. 2008;10:729–40.

5. Giner G, Smyth GK. statmod: Probability Calculations for the Inverse Gaussian Distribution. R J. 2016;8:339–51.

6. O’Toole GA. Microtiter dish biofilm formation assay. J Vis Exp. 2011.
